# Comparing Methods for Mapping *cis* Acting Polymorphisms Using Allelic Expression Ratios

**DOI:** 10.1371/journal.pone.0028636

**Published:** 2011-12-13

**Authors:** Marion Dawn Teare, Suteeraporn Pinyakorn, James Heighway, Mauro F. Santibanez Koref

**Affiliations:** 1 School of Health and Related Research, University of Sheffield, Sheffield, United Kingdom; 2 The HIV Netherlands Australia Thailand Research Collaboration, Pathumwan, Bangkok, Thailand; 3 Cancer Communications and Consultancy Ltd, Knutsford, Cheshire, United Kingdom; 4 Institute of Human Genetics, University of Newcastle, Newcastle upon Tyne, United Kingdom; University of Uppsala, Sweden

## Abstract

Genome wide association studies frequently reveal associations between disease susceptibility and polymorphisms outside coding regions. Such associations cannot always be explained by linkage disequilibrium with changes affecting the transcription products. This has stimulated the interest in characterising sequence variation influencing gene expression levels, in particular in changes acting in *cis*. Differences in transcription between the two alleles at an autosomal locus can be used to test the association between candidate polymorphisms and the modulation of gene expression in *cis*. This type of approach requires at least one transcribed polymorphism and one candidate polymorphism. In the past five years, different methods have been proposed to analyse such data. Here we use simulations and real data sets to compare the power of some of these methods. The results show that when it is not possible to determine the phase between the transcribed and potentially *cis* acting allele there is some advantage in using methods that estimate phased genotype and effect on expression simultaneously. However when the phase can be determined, simple regression models seem preferable because of their simplicity and flexibility. The simulations and the analysis of experimental data suggest that in the majority of situations, methods that assume a lognormal distribution of the allelic expression ratios are both robust to deviations from this assumption and more powerful than alternatives that do not make these assumptions.

## Introduction

**Figure 1:**
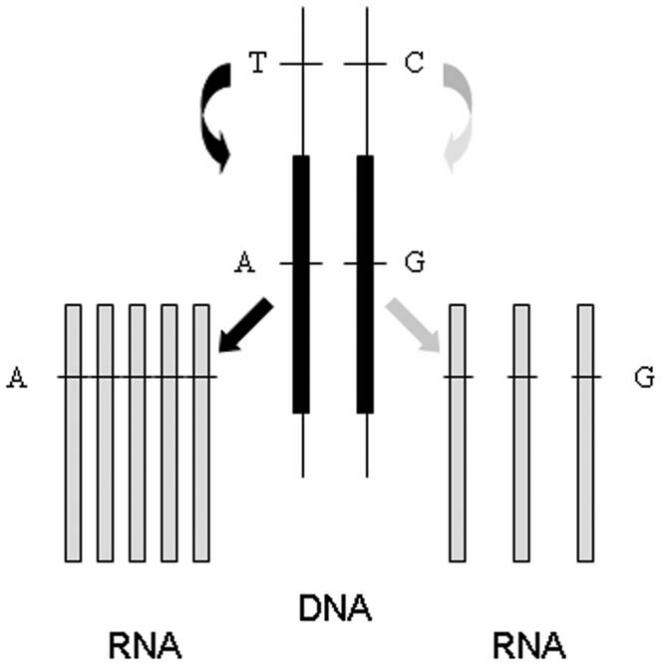
Diagrammatic representation of the effect of a *cis* acting polymorphism upon allelic expression. Depicted is the situation for an individual who is heterozygous for a *cis* acting polymorphism with alleles A and C and is also heterozygous for a polymorphism within the affected transcript.

In recent years, analysis of allelic expression has increasingly been used to ascertain *in vivo* the influence of sequence variants suspected to affect expression in *cis* [1]–[4]. Such variants modulate expression from the same chromosome on which they are located include, for example, changes affecting gene promoters or sequence elements regulating message stability. This is in contrast to factors acting in *trans* that affect transcription of target genes irrespective of their genomic location and whose action is mediated by diffusible components such as transcription factors. Changes acting in *trans* affect both alleles. In individuals heterozygous for one or more transcribed polymorphisms, the contribution of each of the two alleles is assessed by quantifying the relative amount of transcripts from each. Unequal expression designated here as allelic expression imbalance (AEI, also called allele specific expression, ASE, or differential allelic expression, DAE), in individuals heterozygous for a putative *cis* acting polymorphism is seen as evidence for *cis* acting effects. The principle is depicted in Figure 1. It represents an individual heterozygous for a *cis* acting polymorphism with alleles T and C; and a transcribed polymorphism with alleles A and G. This second polymorphism allows us to ascertain the origin of each transcript. The figure shows that transcripts carrying the A allele are more abundant than those carrying the allele G. This is consistent with the T allele of the *cis* acting polymorphism being associated with overexpression (compared to allele C). The rationale behind the use of allelic expression as a tool for mapping *cis* acting polymorphisms is that it should be relatively insensitive to influences affecting both alleles such as sample degradation or *trans* acting effects, compared to methods that analyse expression from both alleles as a pool. Indeed, several recent reports have found that allelic expression analysis can be more powerful in detecting *cis* acting variants than traditional expression quantitative trait locus (eQTL) analysis [1], [2]. This is of particular interest since the effects of polymorphisms may vary between tissues and developmental stages [5], [6], and assessing these effects in tissues where availability is limited will be facilitated by using more sensitive methods of analysis.

**Figure 2:**
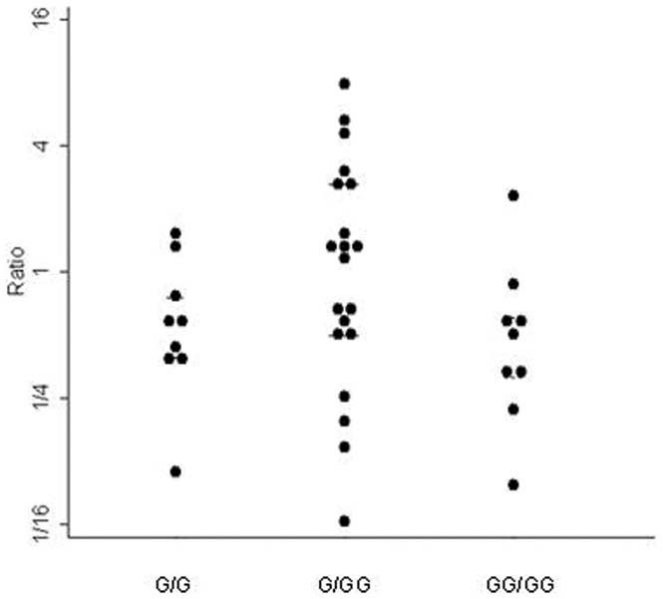
Observed allelic expression ratios measured at rs5854, a transcribed polymorphism at the 3′ end of the MMP1 gene grouped according to the genotype for rs11292517, a polymorphism in the promoter region of the gene.

Allelic expression is often assessed by applying established genotyping methods to cDNA instead of genomic DNA. In a typical experiment DNA and RNA samples are collected from a panel of individuals. We will use here the term sample size for the number of individuals in the panel. The DNA is genotyped for a set of markers that includes at least one marker that is located within the transcript of interest. For individuals heterozygous for the transcribed polymorphism, the RNA, usually after reverse transcription, is used to quantify the relative amounts of transcripts originating from each of the two alleles. The outcomes of such an experiment are the genotype frequencies in the panel of individuals and allelic expression ratios for the individuals that are heterozygous for the transcribed marker. Figure 2 shows an example for such results. Represented are the observed allelic expression ratios measured for a transcribed single nucleotide polymorphism (SNP) at the 3′ end of the MMP1 gene grouped according to the genotype for a polymorphism in the promoter region the gene (Data taken from [7]). The latter is an insertion/deletion polymorphism with alleles G and GG. The data were collected to assess whether the polymorphism in the promoter region is associated with changes in expression in lung tissue *in vivo* [8]. The methods employed to quantify the relative contribution of transcripts from each allele include: restriction fragment analysis [8], DHPLC [9], primer extension using chain terminators and quantification using capillary electrophoresis [5] or mass spectroscopy [10], real time PCR [11], ligations assays [12], [13], or differential hybridisation to oligonucleotide arrays [3], [4], [14]–[17]. Establishing whether an allele is preferentially transcribed requires controls where both alleles are represented in defined proportions. Often, genomic DNA is used as an equimolar control. More recently, transcriptome sequencing has also been used to assess allelic expression levels [18], [19].

Allelic expression can be treated as a qualitative trait, describing the presence or absence of imbalance and perhaps which allele is overexpressed. It can also be considered as a quantitative trait. We will use here the term allelic expression ratio (AER) for the ratio of the signal intensity emanating from one allele, as defined by the transcribed polymorphism, divided by that from the other (e.g. [1], [7], [20]). Alternatives include using the sum of the signal intensities from both alleles in the denominator (e.g. [21]), or consistently using the ratio of signal from the highest expressed allele divided by that from the lowest expressed one (e.g. [22]).

Mapping using allelic expression can be thought of as assessing whether the pattern of imbalance observed across a series of individuals is consistent with a *cis* acting effect for each polymorphism from a set of SNPs. Here we will concentrate on the simplest case where only one candidate polymorphism is tested and only one transcribed polymorphism is used. For an individual that is heterozygous at both the transcribed and the *cis* acting sites, the transcribed allele that is overexpressed will be the one that is on the same chromosome as the as the *cis* acting allele causing overexpression. The phase between alleles at the two sites can vary from individual to individual. Therefore assessing the effect of a putative *cis* acting polymorphism may require determining the phase of the alleles at the transcribed and *cis* acting sites. This is particularly simple when the polymorphism of interest is the transcribed polymorphism itself, resulting in the systematic overexpression of the same allele in heterozygotes, or when both polymorphisms are in complete linkage disequilibrium, where little imbalance will be expected for those homozygous at the *cis* acting site, while those that are heterozygous should show systematic overexpression of the same transcribed allele [5]. In general, when there is less than complete disequilibrium or when the extent of disequilibrium is unknown, there are two possible approaches. The first separates phase estimation from assessing the effect upon transcription. Phase estimation can be done using population data or observing co-segregation of alleles within families. One of the advantages of such an approach is the availability of a plethora of software packages for this purpose (reviewed e.g. in [23], [24]). Once the phase has been taken into account the evidence for the *cis*-acting effect can then be assessed. This analysis can be carried out either using the most likely phased genotype [18], or the estimated distribution of possible phased genotypes [1] for each individual. A second approach is to estimate phase and the *cis*-acting effect simultaneously ([2], [7], [25]).

**Figure 3:**
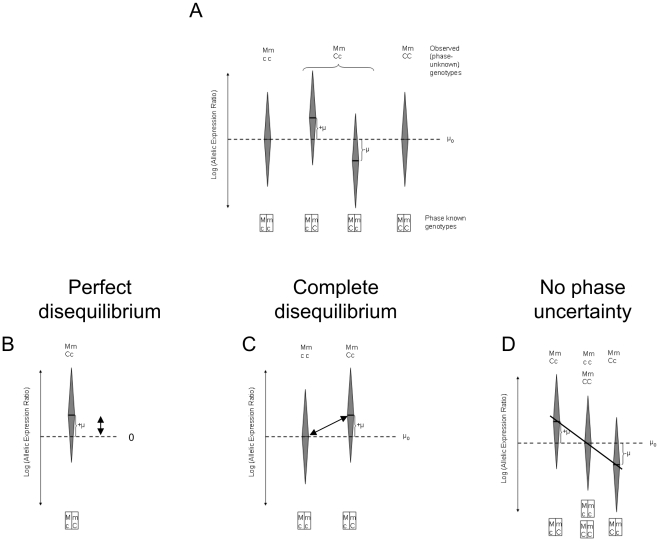
A visualisation of different approaches for testing an association between allelic expression and a biallelic polymorphism. The distribution of allelic expression ratios across a population is represented. We consider here two polymorphisms: a transcribed one, with alleles m and M, used to measure allelic expression; and a *cis* acting one with alleles c and C. Each elongated diamond represents the mean and the spread of the AEI measurements by specific genotypes. A) The general situation. B) Perfect disequilibrium (D′ = 1, R^2^ = 1) between the *cis* acting and the transcribed polymorphism, only two distinct haplotypes exist. C) Complete disequilibrium (D′ = 1, R^2^<1), only three distinct haplotypes exist. D) Situation when the phase between alleles at both sites is known.

For one transcribed and one *cis* acting polymorphism the principles underlying different approaches for testing can be illustrated using Figure 3. These figures include only individuals who are heterozygous for the transcribed SNP, since AER cannot be measured in homozygotes (although the genotype for the putative *cis* acting SNP can vary). The distinct approaches arise due to the extent of linkage disequilibrium present. Panel A represents the relationship between the genotype and allelic expression ratios in the general situation. Panels B to D illustrate the reasoning underlying different tests that have been used. In the simplest case where both polymorphisms are in perfect disequilibrium or the *cis* acting and the transcribed polymorphism are one and the same, testing the effect is consistent with assessing a systematic deviation from balanced expression in one direction (Panel B). Panel C depicts the situation where there is complete disequilibrium. Here the effect of the putative functional polymorphism should result in the systematic overexpression of one and the same transcribed allele in heterozygotes that is not observed among homozygotes. This suggests using a test to assess differences in allelic expression ratios from both groups (e.g. [26]). The situation when the phase is known or can be inferred so that any remaining uncertainty can be neglected is depicted in Panel D. In this case the effect of the putative *cis* acting polymorphism can be assessed by testing the correlation between genotype and ratio, where the genotypes are coded so that the value assigned to homozygotes at the *cis* acting locus is exactly midway between those assigned to the heterozygotes (e.g. [4]).

Nonparametric tests are preferred when there are concerns about sample distribution properties. Their use has been limited to the scenarios presented in Panels B to C, where linkage disequilibrium (LD) is strong (D′ = 1), or Panel D when phase can be confidently inferred.

In order to test the power we simulate the allelic expression. The simplest assumption is to presume that expression from each allele is lognormally distributed. However in practice the patterns observed are more complicated. Several elements contribute to this. Detailed studies routinely uncover that transcription is influenced by more than one polymorphism [1], [27]. In addition we should consider that expression itself is a result of *cis* and *trans* acting factors, therefore although a polymorphism may act in *cis* in a certain context this effect is mediated by trans acting factors that may themselves by the subject of variation caused either by environmental [5], [6], [28], [29] or genetic factors [30]. A related issue is the presence of outliers. This is apparent from the analysis in disease predisposing loci. For example in MLH1 or BRCA1, two genes involved in cancer predisposition, mutations causing nonsense mediated decay lead to a substantial degree of imbalance that overlays the variation due to common polymorphisms. The degree of imbalance in mutation carriers is up to fivefold larger than that observed in samples without mutations (e.g. [25], [31]). Such observations suggest that AEI can be used to identify likely mutation carriers [25] or to assess whether a particular gene is involved in disease predisposition [15]. They also show that rare alleles with a substantial effect on expression can obscure the effect of common alleles.

Here we examine several different but commonly used approaches to the analysis of allelic expression. We focus on the power of different methods to identify sites associated with expression differences in *cis*. We concentrate on the association between allelic expression and particular biallelic polymorphisms and we compare the power using simulated and published data sets.

## Methods

This section has of two parts. In the first part we present the different statistical methods to be compared and in the second we describe and discuss the models used in the simulations. The simulations are used to test the power to detect the effect of a single polymorphism on the AER measured using a single transcribed polymorphism. We consider only biallellic polymorphisms. A significance level threshold of 0.05 is assumed throughout.

*Statistical Tests:* We limit our consideration to previously published approaches or existing methods. These tests assume that the data consist of a set of individuals who have been typed for a putative *cis* acting polymorphism and transcribed marker, and that allelic expression has been measured in those individuals that are heterozygous for the transcribed marker. We use the term sample size to describe the number of individuals genotyped, irrespective of the transcribed marker genotype. The tests can be divided in two groups. The first group relies on a model of the process generating allelic expression. The second group consists of simple statistical tests appropriate for one or more of the instances depicted in Figure 3.

We use the following notation: For the *i*-th individual we designate with *T_i_* the genotyping results for both loci and with *I_i_* the log of the allelic expression ratio. The first set of tests (four in total) are likelihood ratio tests that rely on the assumption that the allelic expression ratios are lognormally distributed with a genotype dependent mean 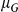 and a genotype independent variance 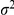. We describe the influence of the genotype and on 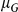 as 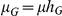, where 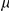 represents the effect of the *cis* acting polymorphism and 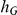 the phase between alleles as both sites, i.e. if we designate with *M* and *m* the alleles at the transcribed site and with *C* and *c* the alleles of the *cis* acting polymorphism then
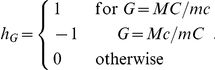
The tests assuming an underlying model differ in the likelihood that is maximised. Test *LRT.j* jointly maximises the parameters describing both expression and haplotype frequencies. The likelihood *L* can be decomposed in two components: 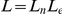. The first is determined by the genotypes of the samples where no allelic expression was measured (this includes the individuals who are homozygous and hence their genotypes for the transcribed marker are MM or mm): 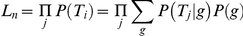, where 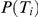 is the probability of the genotyping results, 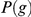 the probability of the genotype *g* given the haplotype frequencies, the index *j* runs through all individuals in the sample for which no AER was measured and *g* through all phased genotypes defined by the two polymorphisms. The second component describes the contribution of samples for which AER was measured 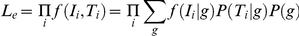, where 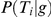, 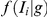, where the index *i* runs through all individuals. This test (*LRT.j*) represents an extension of the procedure described by Teare *et al.* [7] and was used in [25].

The second test we consider is *LRT.p* which maximises the probability of the log expression ratio given the genotyping results :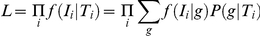. Such a procedure was used in [1]. This method differs from LRT.*j* in that the haplotype frequencies are inferred (or ‘prephased’) from the genotyping results through a preliminary step. For the results presented here this was done here using an Expectation Maximisation (EM) algorithm.

Test *LRT.b* uses instead of the distribution of haplotypes only the most likely haplotype. Thus the likelihood of interest can be described as 
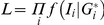
, where 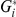 designates the most likely phased genotype for individual *i*. This specific application is equivalent to fitting a simple linear regression.

Test *LRT.k* uses the true or known genotypes instead of the most likely ones. This final LRT test is examined as a gold standard comparison, but in practice the true haplotypes for double heterozygotes are frequently not known.

The second set of procedures we include, rely on some widely used tests, whose application to the analysis of allelic expression is motivated by the considerations discussed in relation to Figure 3. The first approach we investigate consists of using a sign test (S) to assess whether there is a systematic overexpression of one of the transcribed alleles. The sign test uses only the data observed in the single group of individuals who are heterozygous at the putative *cis* acting SNP. The second approach uses the Mann-Whitney or Wilcoxon test (W) to assess whether there is a difference in AER between individuals that are homozygous or heterozygous at the *cis* acting site. In perfect LD (only the double heterozygote group is observed) the test is assumed to fail and a nonsignificant result is returned. These two tests do not assume lognormality of the ratios.

A third procedure investigates whether there is a correlation between AER and the phased genotype (where MC/mc is coded as −1; MC/mC or Mc/mc as 0; and Mc/mC as 1). This requires assigning one phased genotype to each individual. To apply the test in the case of haplotype phase uncertainty (C) the heterozygous individuals are assigned the most likely phase, resulting in only one heterozygote group. We compare the results using the same test but using the true or known simulated genotypes (C.k).

A fourth possibility we investigate is to compare the variance of AER between homozygous and heterozygous at the *cis* acting site using an F-test (V). This test would appear most suitable under linkage equilibrium (see Figure 1 A).

While it is unlikely that the haplotype phase would be known, we show the results of applying some methods to known phase data to see the loss in power due to lack of information.

A summary and overview of the tests used is presented in Table 1.

**Table 1:** Summary of tests used.

Test	Motivation^a^	Advantages	Disadvantages	Notes
**LRT** (Likelihood Ratio Tests)	General situation	Easy to expand (e.g. several cis acting sites).	Assumption of Log normality.	Assume that expression from one allele is drawn from a lognormal distribution
**LRT.j** (joint)			Requires specialised software	Enables joint estimation of phase and effect.
**LRT.p** (prephased)			Compared to LRT.j reduced power in the absence of disequilibrium	Two step procedure: In the first step the phased genotype probabilities are estimated and in the second the effect is assessed.
**LRT.b** (most likely genotype , “best”)		Simple calculation	Lack of power when phase uncertain	As LRT.p but uses the most likely (best) phased genotype for each individual For R^2^<1 corresponds to regression of the log AER onto the most likely genotype.
**LRT.k** (known genotype)				As LRT.b but uses true simulated genotype. Represents the outcome of the LRT tests once phase uncertainty has been eliminated.
**S** (Sign)	Perfect disequilibrium (R^2^ = 1)	No assumption on distribution	Diminishing power when SNPs tend to equilibrium	Tests systematic overexpression of one of the alleles. We use here the Sign test.
**V** (Variance)	Linkage equilibrium (|D′| = 0,R^2^ = 0)	Does not require estimating phase	Diminishing power with increasing disequilibrium. Assumes lognormality^b^	Tests whether the spread of AER is larger among heterozygous at the *cis* acting locus than among homozygous. We use here an F-test for the comparison
**C** (Correlation)	|D′|<1, R^2^<1.	Insensitive to transcribed marker effect	Lack of power when phase uncertain. Assumes lognormality^b^	Requires at least two distinct genotypes to be observed at the cis acting site among transcribed marker heterozygotes. Assumes that the phase can be inferred in double heterozygotes, so we use here the most likely genotype.
**C.k** (Correlation , known genotype)	|D′|<1, R^2^<1.			Represents the outcome of the test above once phase uncertainty has been eliminated.
**W** (Wilcoxon)	Complete disequilibrium (|D′| = 1,R^2^<1)	No assumption on distribution	Assumes that all double heterozygotes have the same phased genotype	Tests whether there is a difference in AER between heterozygotes and homozygotes for the cis acting polymorphism. We use here the Wilcoxon test.

a : Pattern of disequilibrium, as represented in Figure 3, for which the test is most appropriate.

b : Assumes that given the genotype AERs follow a log normal distribution.

*Simulations:* The data were simulated under four basic models. This allowed us to explore the effects of different parameter values as well as different assumptions concerning the processes modulating allelic expression. In all our simulations we assume that the variance of the expression from one chromosome is independent of its genotype and from expression levels and that the effects of different polymorphisms combine in a multiplicative manner, i.e. for a set of *K cis* acting polymorphisms with effects 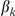 (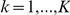) the expected log of the allelic expression ratio in a sample is 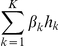 where 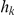 describes the phase between the alleles at the k-th *cis* acting polymorphism with respect to the transcribed marker allele in this sample.

The first set of simulations assumes that allelic expression is influenced only by a single biallelic site and that for each allele expression can be described by a lognormal distribution. The model can be described by five parameters: three haplotype frequencies, the expected log ratio of expression from the one of the *cis* acting alleles divided by that from the other allele, and the variance.

The second set of simulations considers the commonly encountered situation when one of the transcribed alleles is overexpressed and investigates the power to detect the effect of a second *cis* acting polymorphism. Such consistent overexpression of one of the transcribed alleles is often reported (e.g. [20]). This can be a consequence of *cis* acting polymorphisms in perfect disequilibrium with the transcribed marker or can reflect problems with the normalisation to equimolar controls. This model includes a parameter 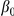 which describes the mean overexpresssion of one of the transcribed alleles relative to that of the other. In our model this corresponds to the expected ratio for the homozygotes at the *cis*-acting candidate.

The third set of simulations allows expression to be determined by several sites. We assume that we are investigating the site with the largest effect, that expression is influenced by a number of sites, that the effect size follows an exponential distribution, that all effects are mediated by biallelic polymorphisms and that the additional sites are in linkage equilibrium with both the transcribed and our test polymorphism. We further assume that the allele frequencies at all of the *cis* acting loci are equal to 0.5 and that there is no effect from the transcribed polymorphism. We expand the first model to include the *n* additional *cis* acting sites. We first simulate the phased genotypes of K individuals for *n+2* biallelic loci. The minor allele frequencies for the first two markers are p_M_ and p_C_ and for the remaining ones 0.5. The *n+2* markers are all in linkage equilibrium. The algorithmic form of the simulations is as follows: we sample *n+1* values, 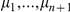, from an exponential distribution with a rate of 0.1. These values are then divided by that with the largest value 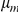 and multiplied by 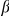, i.e. 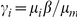, to ensure that the largest effect is 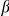, this effect is attributed to the candidate *cis* acting locus. We then simulate for each individual the log ratio 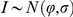, where the mean 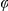 is dependent upon the genotype: 
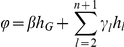
 , 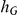 characterises the phase between alleles at the transcribed and the main *cis* acting site and 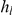 the phase between the transcribed and *l*-th *cis* acting marker given the genotype of the individual.

A fourth set of simulations considers the effects of outliers. We explore here the situation that arises when the AER of some individuals appears to be drawn from a distribution different from the one described by the simple model used for the first set of simulations. We assume that this is caused by the presence of certain alleles which we call outliers. Irrespective of the genotype at the *cis* acting site the logarithm of the allelic expression ratio for an individual that is heterozygous for the transcribed marker and carries one outlier allele is normally distributed with a mean 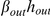 and a variance 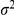, where 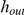 describes the phase between outlier allele and transcribed marker. In the case when the both alleles are outliers with respect to expression the mean log AER was *1*. This requires two additional parameters: one describing the mean effect of the outlier 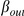 and a second describing the frequency of outlier alleles 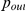.

In the final simulation scenario we assume that the log of the allelic expression is not normally distributed but can be described by a heavy tailed distribution; we use here a t-distribution with two degrees of freedom.

*Published data:* We finally demonstrate the power of the various methods in real data situations by using two previously published data sets (Table 2). In these two examples there is experimental evidence for the *cis* acting effect of the nontranscribed SNPs. The two datasets used in this study have been previously published and details including recruitment, sample collection and ethical approval can be found in the original publications [8], [25]. The first set consists of data from individuals typed for a transcribed polymorphism in the 3′ untranslated region of the matrix metalloproteinase I gene (*MMP1*), that were also typed for a polymorphism in the promoter [7], [8]). Reporter assays have shown that this polymorphism can modify transcription *in vitro* [32]. The individuals in the second set were assessed for a transcribed polymorphism in the MLH1(mutL homolog 1) gene [25]. The samples were also genotyped for a marker in the 5′ region of the gene, that has been recently been shown to influence transcription *in vitro* [33]. The influence of sample size and analysis method on power was assessed by sampling from the observed datasets with replacement.

**Table 2:** Experimental data sets.

Data set name	*MMP1*	*MLH1*
References	[7], [8]	[25]
Genotyped Individuals	107	257
Transcribed SNP	rs5854	rs1799977
*Cis* acting SNP	rs11292517^a^	rs1800734^b^
AER		
Individuals analysed	38	74
Method	RFLP and gel densitometry	MALDI-TOF
Comments		Samples affected by non-sense mediated decay have been excluded

a : [32].

b : [33].

## Results

**Figure 4:**
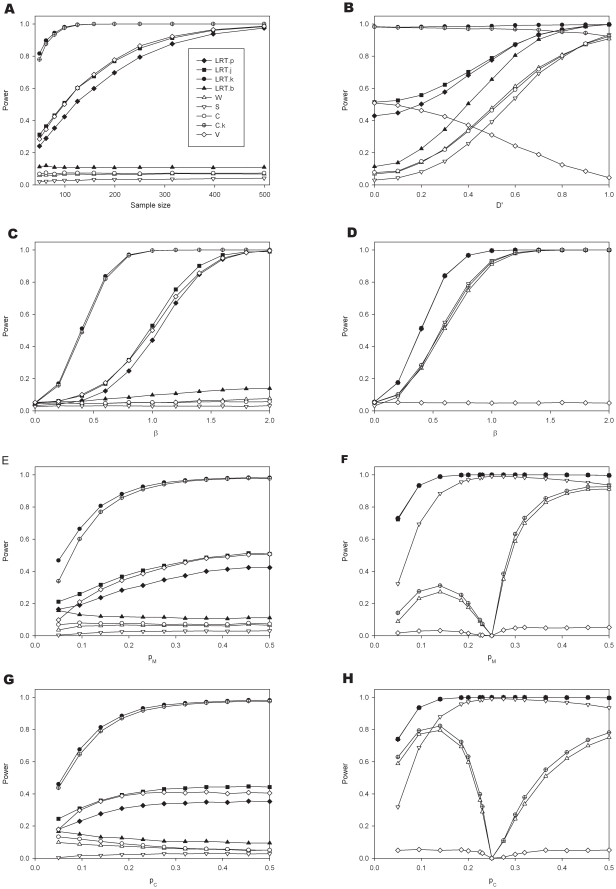
Power comparisons when data are simulated assuming a log normal distribution for the allelic expression ratios. For all simulations: 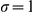. Panel A: Effect of sample size assuming transcribed and *cis* acting polymorphism are in linkage equilibrium (Simulation parameters: 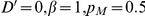 and 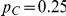 ). Panel B: The influence of the extent of disequilibrium (Simulation parameters: 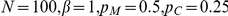); Panels C and D: The influence of effect size (Panel C for 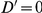 and panel D for 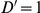 other simulation parameters 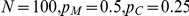). Panels E and F: The influence of allele frequency for the transcribed polymorphism (Panel E for 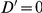 and panel F for 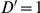, othersimulation parameters: 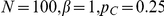). Panels G and H: The influence of allele frequency for the *cis* acting variant (Panel G for 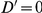 and Panel H for 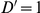,other parameters: 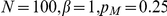).

**Figure 5:**
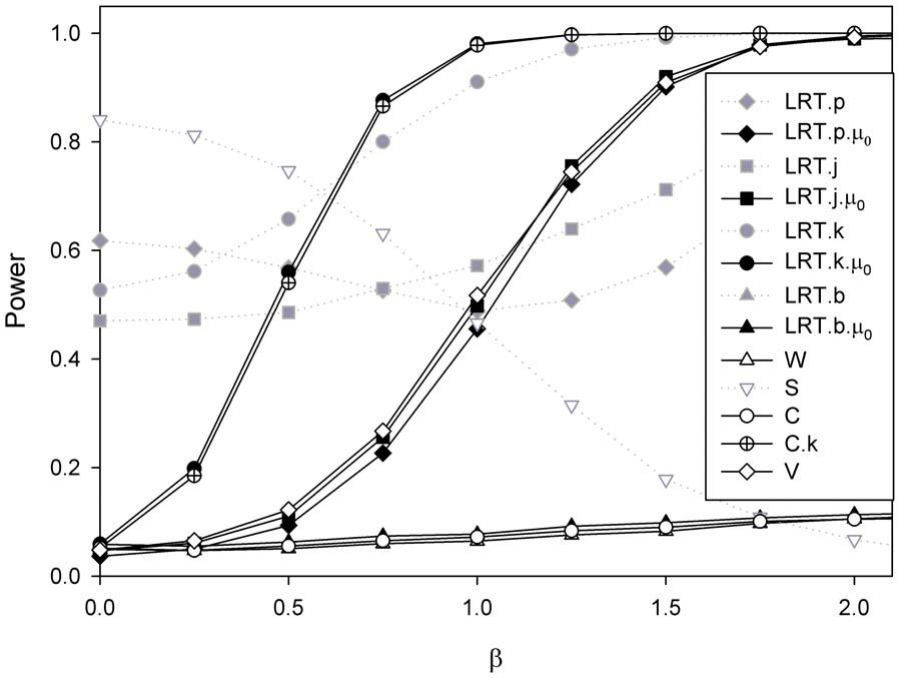
Power comparisons when the simulated model allows for one transcribed marker allele to be consistently over-expressed. Simulation parameters: 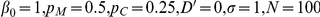. Analysis in greyscale is conducted using (misspecified) methods that do not allow for an allele specific expression effect from the transcribed polymorphism (Panel A: 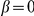, i.e. no effect from the *cis* acting polymorphism, and panel B: 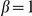). Panels C and D: Analysis conducted using models that do allow for an effect from the transcribed polymorphism (Panel C: 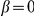 and panel D: 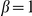).

**Figure 6:**
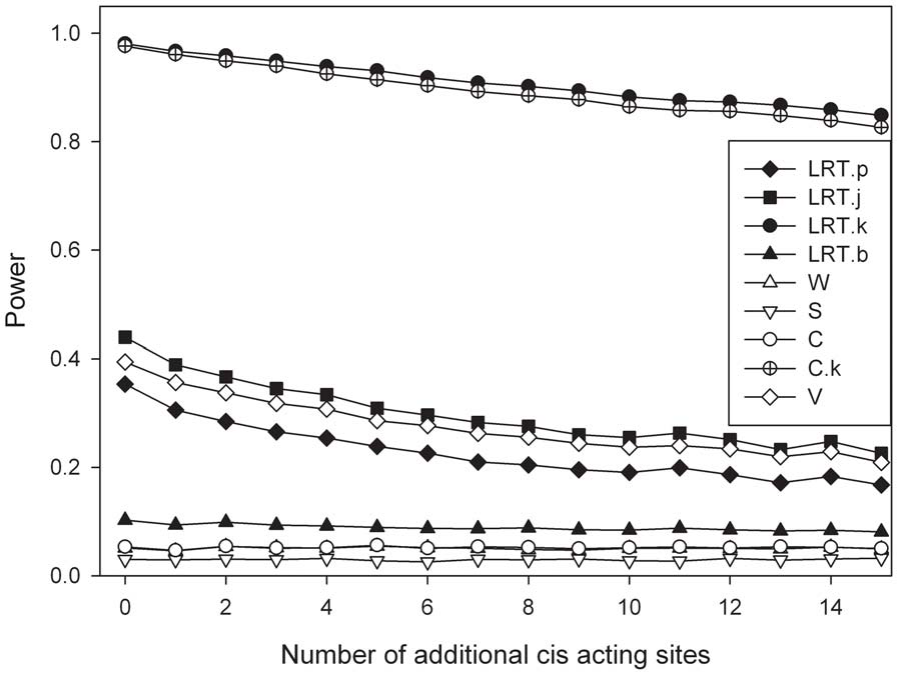
Additional sites affecting the expression in *cis*. The graph represents the influence of the number of sites upon the power to detect the SNP with the largest effect. All polymorphisms are assumed to be in linkage disequilibrium. Simulation parameters: 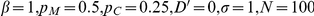.

**Figure 7:**
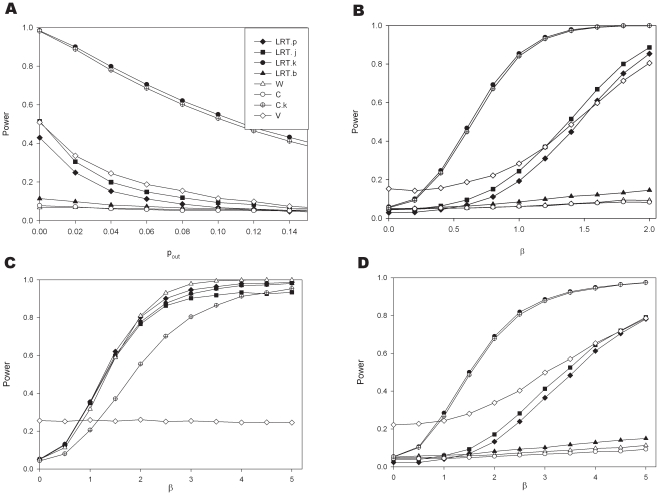
Deviation from a simple log normal distribution (Simulation parameters 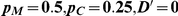). Panels A and B show the effects of outliers (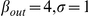). In panel A 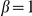 and in Panel B the outlier frequency, *p_out_*, is 0.03: Panels C and D present the situation when the log of the expression of each allele follows a t-distribution with 2 degrees of freedom (C for 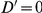 and D for 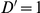).

**Figure 8:**
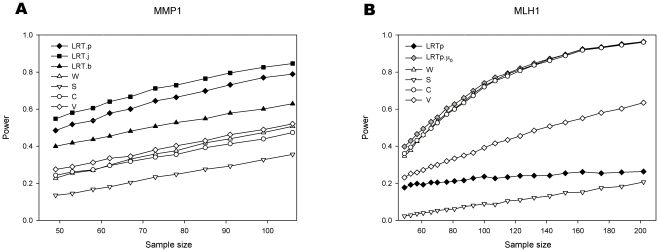
Effect of sample size in experimental data. We examine here the power to detect the *cis* acting effect of polymorphisms known to affect transcription for MMP1 (panel A) and MLH1 (panel B).

The results are summarised and presented in five figures. Each investigates the power of the different tests when conditions such as sample size, extent of LD or allele frequency are permitted to vary. The first four figures use simulated data. First we assess the power of the different tests when AER simply follows a log normal distribution (Figure 4), then we investigate the case when one transcribed allele is consistently overexpressed and we wish to asses an independent effect of the cis acting polymorphism (Figure 5). The situation when there are additional sites affecting expression is investigated in Figure 6. Figure 7 explores deviations from the lognormal distribution. In the final figure (Figure 8) we use previously published experimental data to assess the impact of sample size.

Figure 4 explores the effects of varying parameter values using the simplest simulation model, i.e. one *cis* acting polymorphism and a lognormal distribution for the allelic expression ratios. In panel A the transcribed and *cis* acting polymorphisms are in linkage equilibrium (D′ = 0). The effect of sample size on the power to detect an association with the seven different methods can be seen. The joint phase and effect estimation (LRT.j) performs better than methods where the haplotypes are estimated first and the effect assessed in a separate step. However, its performance is strongly affected by the extent of disequilibrium between both markers. Panel B shows for a sample size of 100, that although there is a difference between both types of methods (LRT.j and LRT.p) the advantage diminishes quickly with increasing disequilibrium. It should also be noted that the most likely phase test (LRT.b) achieves a comparable power to LRT.j for modest disequilibrium (D′ = 0.5). The variance test works very well when D′ = 0 however as D′ increases this test only reduces in power whereas all the other tests improve.

Changes in effect size are explored in panels C (for D′ = 0) and D (D′ = 1). Where there is no effect (i.e. β = 0) the type I error seems adequately controlled. Under linkage equilibrium the LRT.j is slightly more powerful than LRT.p. While the test using Pearson's correlation coefficient (test C) works very well when phase is known, the test is ineffective when haplotype uncertainty exists. The effects of changes in the allele frequency for the transcribed polymorphism are explored in panels E and F, and of the putative *cis* acting in panel G and H. The power increases with the proportion of heterozygotes for the transcribed markers that are also heterozygotes for the *cis* acting variant. Therefore in the absence of disequilibrium (D′ = 0) power increases with heterozygosity (panels E and G), while for D′ = 1 a maximum is achieved in the presence of matching allele frequencies for the model based tests (perfect disequilibrium, R^2^ = 1, panels F and H). In panels F and H we see the drop in power of the two tests C.k and W as the system approaches perfect disequilibrium, reflecting the fact that when there is perfect disequilibrium all heterozygotes have the same genotype.

Figure 5 considers the case when there is an effect on transcription associated with the transcribed polymorphism itself. The methods tested assume either that we are unaware of an effect associated with the transcribed polymorphism and therefore use tests that do not explicitly consider such an effect (LRT.p, LRT.j, LRT.k. LRT.b, W,S,C, C.k and V) or that we are aware and employ tests that allow for the effect (LRT.p.μ_0_, LRT.j.μ_0_, LRT.k.μ_0_ and LRT.b.μ_0_). The lines depicted in grey present analyses in which the type I error rate is not adequately controlled. The sign test S uses only the AER observed on heterozygotes so this test will be affected by the transcribed SNP effect. If analysis is conducted using the LRT approach but the β_0_ parameter is neglected then these methods are compromised. Therefore we used a set of tests (LRT.p.μ_0_, LRT.j.μ_0_ , LRT.k.μ_0_ and LRT.b.μ_0_), that allow for an effect of the transcribed polymorphism itself. Here the expected log AER is described as 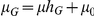, where μ_0_ represents the effect of the transcribed polymorphism. The LRT.j and LRT.p both perform well and much better than the LRT.b and the correlation test (C).

Figure 6 presents the results for the scenario of multiple *cis* acting loci, though there is now no effect of the transcribed marker itself. The simulations constrain the effect of the candidate SNP to be the strongest effect. As should be anticipated power reduces with increasing additional causative loci. This figure assumes that D′ = 0.

Figure 7 explores the effects when the AER distribution deviates from a simple lognormal distribution. Panel A presents the effects of outliers. It shows that with increasing outlier frequency the power is quickly lost. As expected, tests relying on a nonparametric method to assess the influence of the *cis* acting polymorphisms are affected to a lesser degree. With increasing outlier frequency the performance of the LRT.p and that of the LRT.j method becomes similar. The presence of outliers does not affect the ability to determine haplotype frequencies in the former. The power of the variance test appears to be higher than the LRT methods. However, this is accompanied by an inflated type I error as can be seen in panel B when the true effect size is zero. Panels C and D show that when the log of the expression of each allele follows a t-distribution with 2 degrees of freedom, there is a substantial loss of power in particular for the model based methods where the phase needs to be estimated.

Figure 8 shows the analysis of real data using two previously published data sets (see Table 2 for details). In both cases there is experimental evidence for the *cis* acting effect of the nontranscribed SNPs. The Figure explores the effect of varying sample size. These two examples differ in two aspects. The first is that in *MMP1* (panel A) the *cis* acting and transcribed markers are close to linkage equilibrium (D′ = 0.05, R^2^ = 0.00) while in *MLH1* (panel B) they are in strong disequilibrium (D′ = 0.99, R^2^ = 0.09). Therefore we see differences in the power attained by the LRT.j, LRT.p and LRT.b tests for *MMP1* (panel A), while for *MLH1* the curves for these same tests are indistinguishable. The second difference is that while there is no evidence for a transcribed marker associated effect for *MMP1* [7], there is systematic overexpression of one of the transcribed alleles for *MLH1* [25]. This effect is such that for double heterozygotes the allele associated with overexpression at the *cis* acting polymorphism is predominantly occurring in phase with the underexpressed transcribed marker allele. So those tests that do not allow for a β_0_ parameter will perform very poorly in this situation.

## Discussion

The results demonstrate that phase uncertainty is the main factor determining the power of the tests. However, closer inspection of the Figures shows that this effect depends on the allele frequencies at both sites. In the absence of disequilibrium, i.e. when the two loci are not associated (R^2^ = D′ = 0), power increases with increasing heterozygosity at both the transcribed and the *cis*-acting sites (Figure 4, panels E and G). When. there is no phase uncertainty in double heterozygotes, i.e. in the case of complete LD, the power of the likelihood ratio tests and that of the procedure based on the sign test, peak when both markers have the same minor allele frequency, i.e. are in perfect disequilibrium (R^2^ = 1).

One important consequence of the influence of extent of disequilibrium on power is that the effects of polymorphisms that are physically closer to the transcribed marker will be easier to detect than those of more distant markers, since disequilibrium is expected to be weaker for the latter. Perfect disequilibrium between transcribed and *cis*-acting polymorphism is equivalent to the situation when there is an effect on transcription associated with the transcribed polymorphism itself. However technical artefacts such as problems with normalisation to equimolar controls can also lead to assigning an effect to the transcribed polymorphism.

We explored the situation where the transcribed polymorphism influences transcription and we wish to assess an additional effect associated with a second polymorphism. In this case, likelihood ratio tests that do not include a baseline term (μ_0_), have an inflated type I error, wrongly attributing an effect where there is none. This can be circumvented by using a test that allows for an effect associated with the transcribed polymorphism. However the evidence for the second effect becomes difficult to obtain when the polymorphisms are closely associated.

The presence of outliers quickly degrades the ability to detect *cis* acting sites. Since outliers distort the distribution of allelic expression ratios, it is not surprising to observe that a nonparametric method such as that relying on the Wilcoxon test, is less affected. With increasing outlier frequency the performance of the LRT.p and that of the LRT.j method become similar. This is consistent with the fact that the presence of outliers does not affect the ability to determine haplotype frequencies in the former, while a misspecified model will impair estimation of haplotype frequencies in the latter. However, the performance of the joint estimation method does not drop below that of the method where allele frequencies are estimated without using expression information. Similar observations can be made when there are several *cis* acting loci and where the distribution of expression is heavy tailed. Also in these cases the allelic expression ratios given the genotype at the *cis* acting and transcribed polymorphism do not follow a lognormal distribution.

Throughout our simulations the test relying on joint maximisation of effect and haplotype frequencies is more powerful than the test where the genotypes are estimated separately in a first step and this is in turn more powerful than the a test were only the most likely phase is used. This difference is substantial for the *MMP1* data where for a sample size of 105 the first test has 85% power, while the second 70% and the third 65% (Figure 8 panel A). However the difference between the first two methods quickly disappears with increasing disequilibrium. There is no discernable advantage of any of the three methods for the *MLH1* data. Joint determination of phase and effect is more cumbersome than using the predetermined haplotype frequencies, a task for which a wide range of tools has been developed over the past fifteen years. Indeed, a dense enough panel of typed markers may eliminate most of the haplotype uncertainty, leading to situations where testing can be done using linear regression (see Figure 3 panel D). This opens the way for using standard statistical packages to assess more complex models including several *cis* acting polymorphisms and other co-variates. In our simulations and analysis the model LRT.b is equivalent to fitting simple linear regression.

Another conclusion from our data is that for the range of situations studied the advantage gained by using tests that dispense from the lognormality assumption is at best slight and led, in the majority of the simulations scenarios and in the real data sets used, to a substantial loss of power in the ability to detect experimentally supported polymorphisms acting in *cis*. The variance test that groups the homozygotes and heterozygotes together is a powerful test when linkage equilibrium exists, though power reduces as disequilibrium increases. However, this test gives an inflated type I error in the presence of outliers and extreme values.

In summary our investigation shows that when it is not possible to determine the phase between the transcribed and potentially *cis* acting allele there is generally some advantage in using methods that estimate genotype and effect on expression simultaneously. However when the phase can be determined, simple regression models seem preferable. The scenarios explored here by simulation and through experimental data show that methods assuming lognormal distributions are the most powerful and are generally robust with respect to presence of outliers and other deviations from lognormality.
